# Supplementary material for: Seismic evidence for melt-rich lithosphere-asthenosphere boundary beneath young slab at Cascadia
Source: Nat Commun. 2024 Apr 25;15:3504. doi: 10.1038/s41467-024-47887-0 (PMC11045719; doi:10.1038/s41467-024-47887-0)
Supplement: Supplementary file 1 — Supplementary Information [file 41467_2024_47887_MOESM1_ESM.pdf]

Supplementary Information for

Seismic evidence for melt-rich Lithosphere-Asthenosphere Boundary beneath young slab at Cascadia

Xin Wang[1,2,3], Ling Chen[2,4*], Kelin Wang[5], Qi-Fu Chen[1,2], Zhongwen Zhan[3], Jianfeng Yang[2,4]

[1] Key Laboratory of Earth and Planetary Physics, Institute of Geology and Geophysics, Chinese Academy of Sciences, Beijing, China.

[2] College of Earth and Planetary Sciences, University of Chinese Academy of Sciences, Beijing, China.

[3] Seismological Laboratory, California Institute of Technology, Pasadena, CA. USA.

[4] State Key Laboratory of Lithospheric Evolution, Institute of Geology and Geophysics, Chinese Academy of Sciences, Beijing, China.

[5] Pacific Geoscience Centre, Geological Survey of Canada, Sidney, BC, Canada.

[*]Corresponding to: Ling Chen (lchen@mail.iggcas.ac.cn)

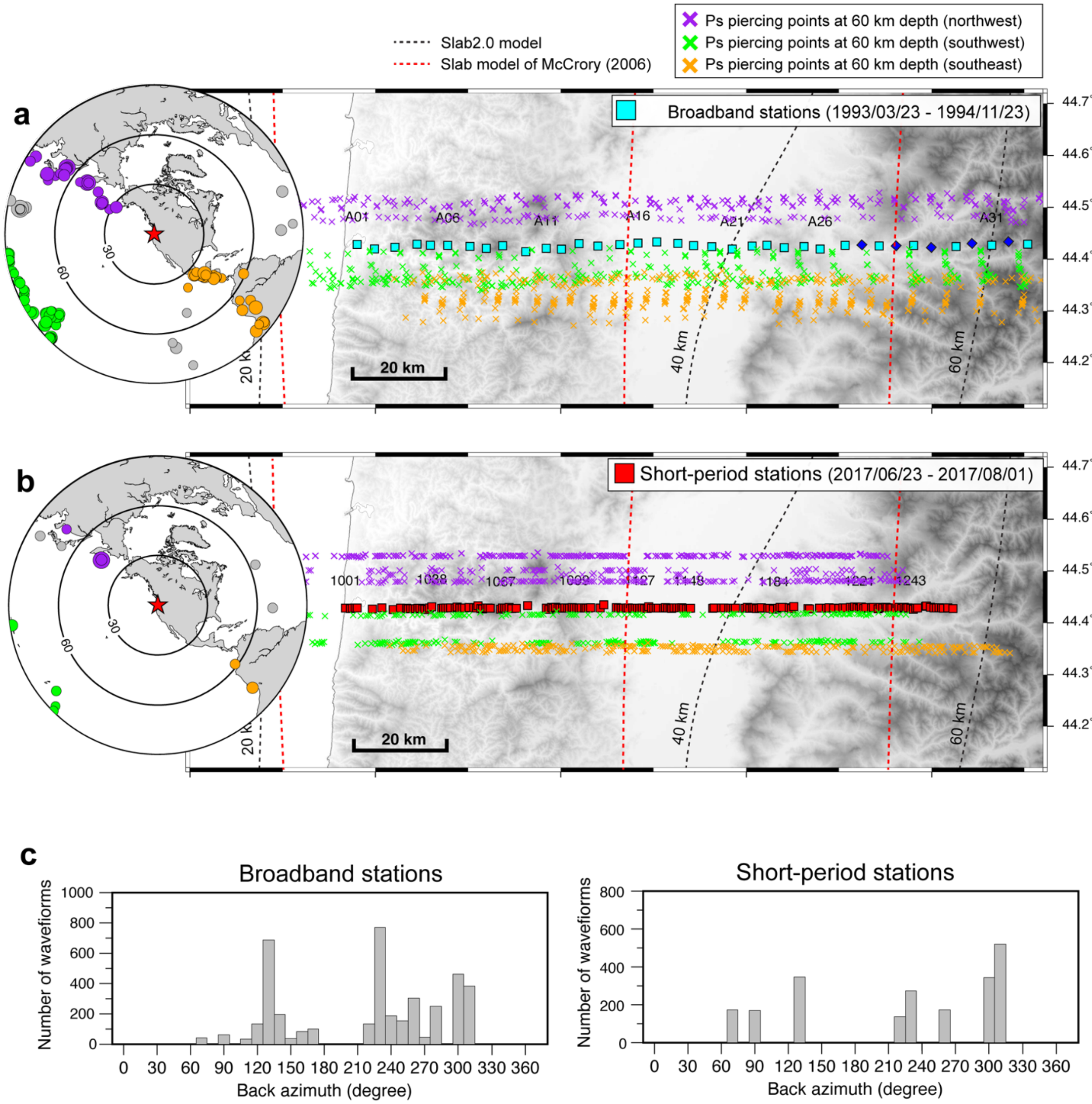

Fig. S1 | Location of seismic stations and earthquakes used for Coherent Receiver Function (CRF) imaging in this study. a Broadband seismic stations (local map) and teleseismic events (M>5.8) recorded during their observation period (world map). Ps piercing points (crosses) at 60 km depth, color coded to show back-azimuths. Events in the three shown directions are used to obtain the results shown in Fig. S3. The maps were generated using Generic Mapping Tools[1], with topography data from the Global Multi-Resolution Topography Synthesis[2]. The black and red dashed lines represent depth contours of the slab interface model with data from Slab2.0[3] and McCrory et al.[4], respectively. **b** Similar to (a) but for the short-period nodal seismic stations. **c** Histograms show the distribution of teleseismic waveforms after data pre-processing across different back azimuths.

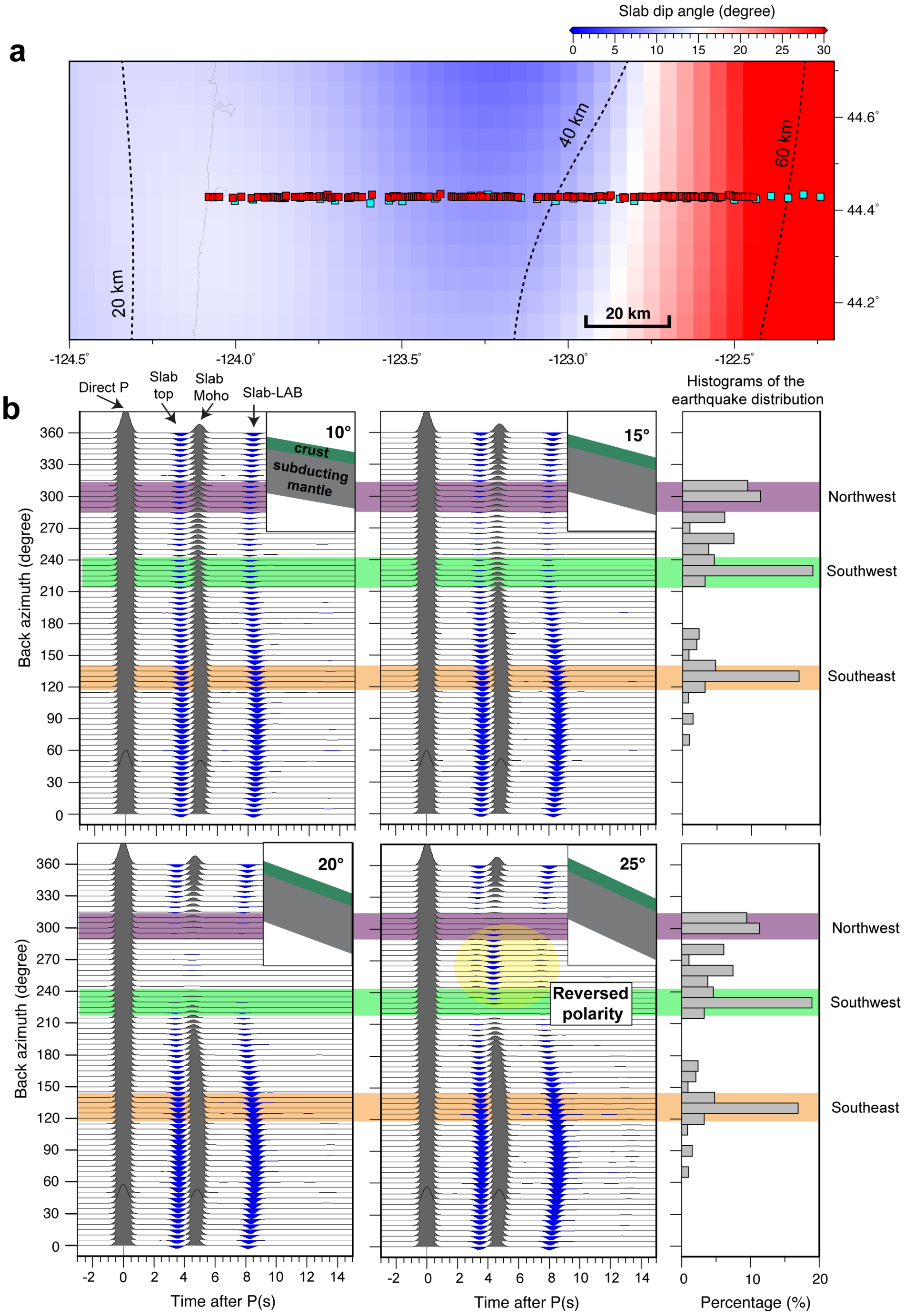

Fig. S2 | Effect of the eastward dip direction of the Juan de Fuca plate on Receiver Function (RF) analyses. a Slab dip angle from the Slab2.0 model[3]. **b** Synthetic RFs for different slab dips as shown in upper right. The amplitudes of Ps conversions vary with the back azimuth. Note the polarity reversal of westerly back azimuths in the dip=25° model. The histogram shows the number of selected telseismic waveforms in different back azimuths. The three horizontal strips highlight the three groups of earthquakes in the northwest, southwest, and southeast directions shown in Fig. S1.

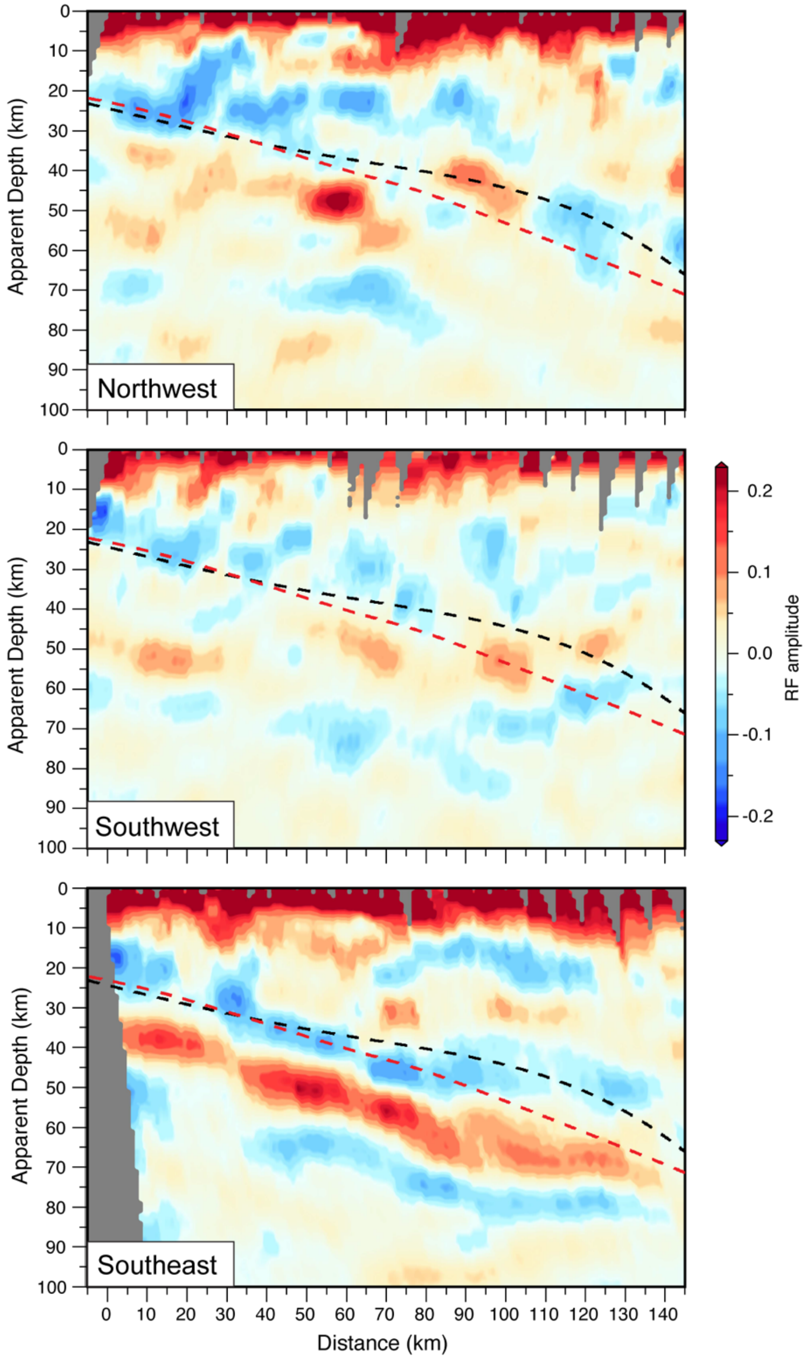

Fig. S3 | Conventional single-station Receiver Function (RF) deconvolution and stacking results for waves from different directions. Here, the RF images are obtained using broadband data filtered in 0.15 to 1.0 Hz for demonstration purposes. See Fig. S1 for locations of teleseismic events. The black and red dashed lines represent depth contours of the slab interface model from Slab2.0[3] and McCrory et al.[4], respectively. Because the Juan de Fuca slab dips roughly towards the east, the waveforms from the southeast are more effective in constraining the slab-parallel seismic discontinuities, compared with those from the other two directions.

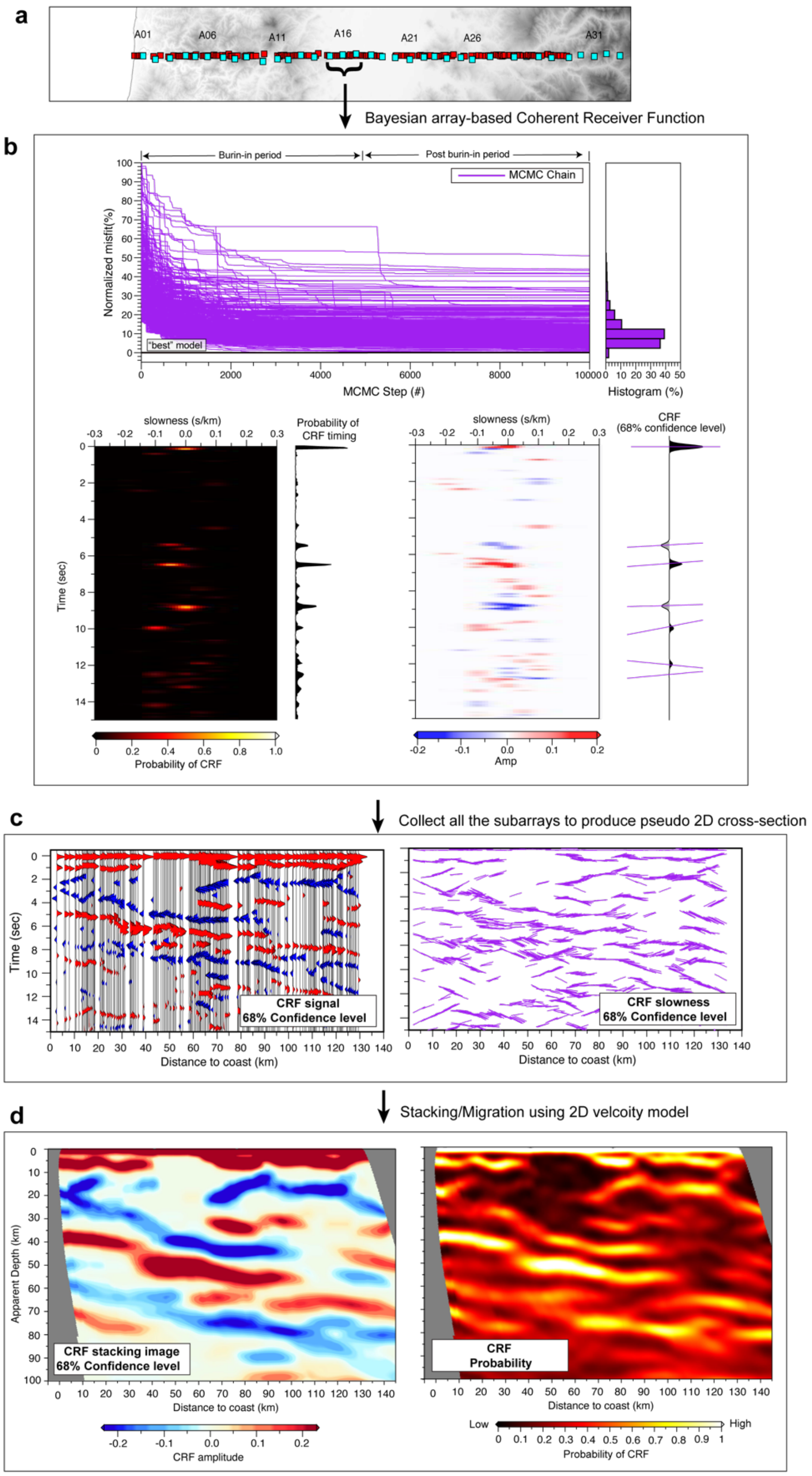

Fig. S4 | Workflow for Bayesian array-based Coherent Receiver Function (CRF) imaging. a The locations of seismic stations used in this study. We used a moving window of ~15 km, generally including 3 broadband stations and 25 short-period stations, to conduct the CRF

analyses. **b** Bayesian array-based CRF deconvolution. In each subarray, the subsurface structure is parameterized as the number of phase, the timing, amplitude, and slowness of each phase. The upper panel shows relative misfit (normalized to the "best" model) against iterations for 500 chains running in parallel. Each chain contains 10,000 Markov chain Monte Carlo (McMC) iterations, with the second half of iterations used to generate the ensemble solutions. The histogram shown in the right panel shows the distribution of misfit for all the ensemble solutions. For the lower panel, from the left to the right, these sub-panels show the density plot of the ensemble solutions that represents the posterior probability distribution of a CRF signal at a given time and slowness, the probability distribution of the timing of CRF phases (or the layering structures), the CRF amplitude as a function of timing and slowness, and the 68% confidence level 1D CRF for this sub-array. **c** The pseudo 2-D cross-section by simple juxtaposition of individual CRFs obtained in each sub-array and the corresponding slowness information. **d** The CRF stacking images. The left panel only shows the CRF signals with a 68% confidence level, while the right panel shows the probability of CRF signals. Here, the CRF results are obtained using both broadband and short-period data filtered in the 0.15 to 1.0 Hz range for demonstration purposes.

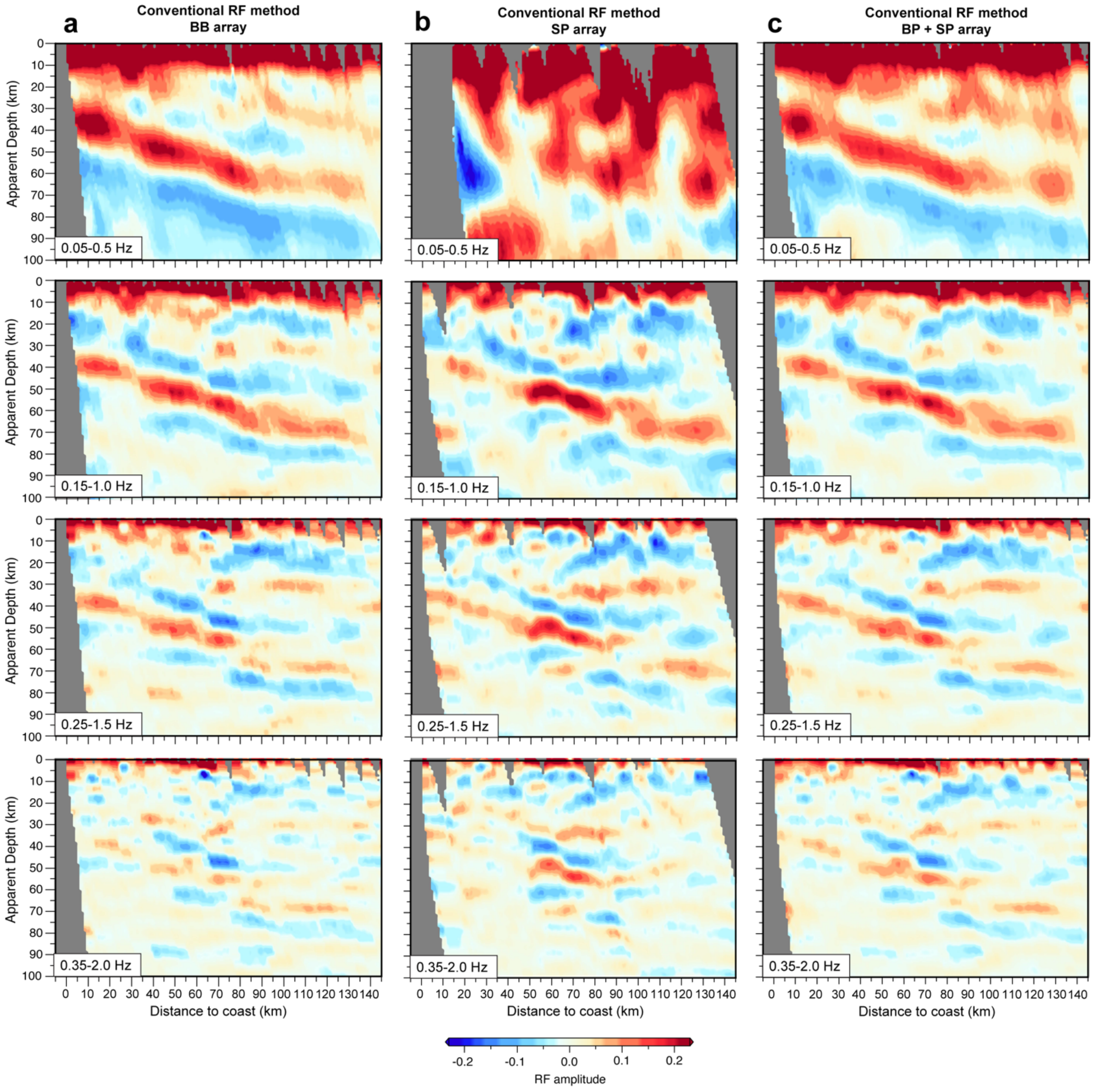

Fig. S5 | Conventional Receiver Function (RF) images. a RF images constructed using broadband data with waves from southeast. See Fig. S1 for locations of teleseismic events. From top to bottom, panels show results obtained for different frequency bands. **b** Similar to (a), but for RF images obtained using short-period data. Due to the narrow frequency band of short-period seismic stations, the 0.05-0.5 Hz RF stacking image is not reliable. **c** Similar to (a), but for RF images obtained using broadband and short-period data. It is important to note that the negative phase (at depths around 20-40 km) above the slab Moho is disappeared in the 0.05-0.5 Hz conventional RF images, due to the strong effects of the slab Moho Ps phase.

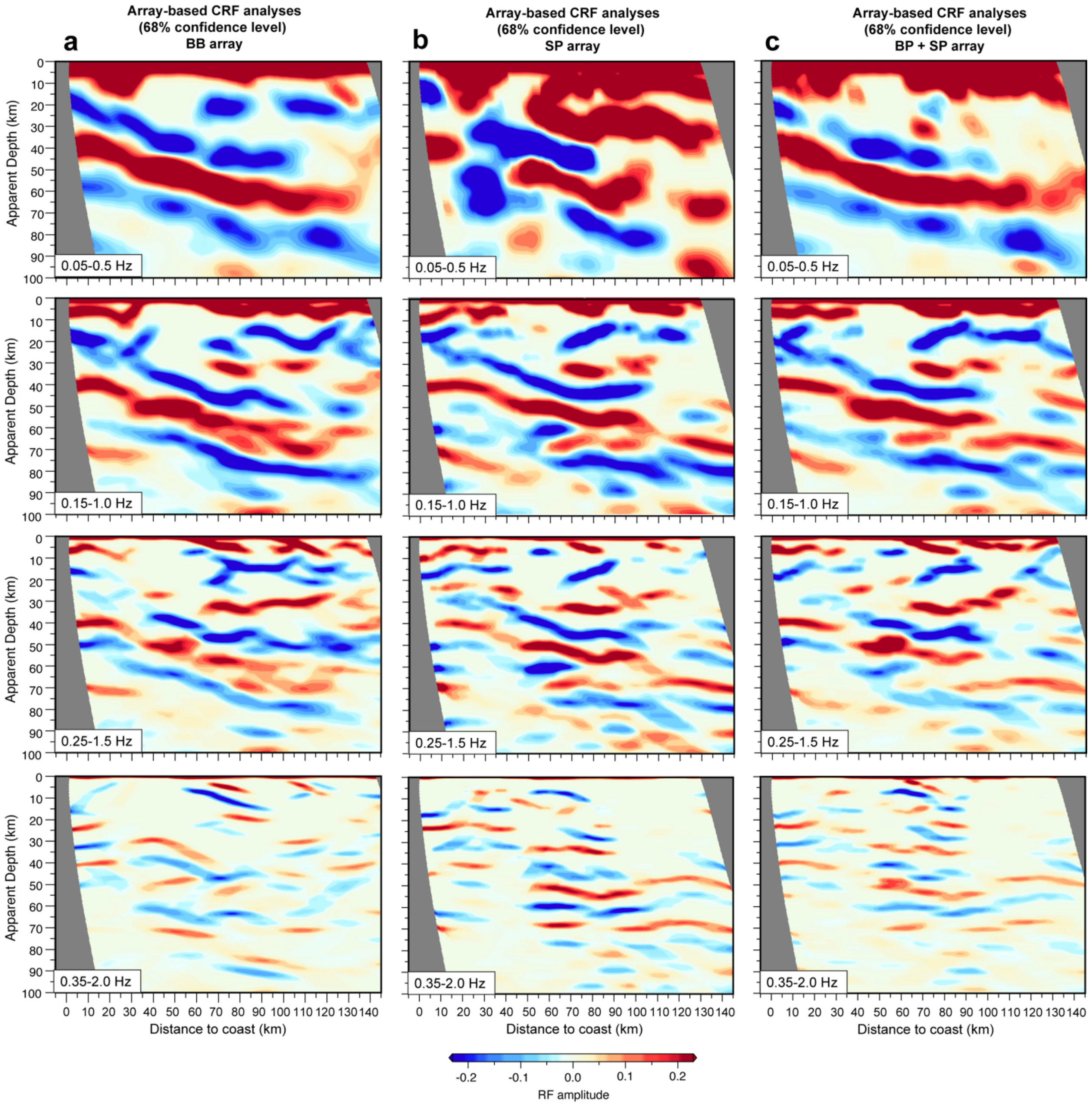

Fig. S6 | Bayesian array-based Coherent Receiver Function (CRF) images. a CRF images constructed using broadband data with waves from southeast. See Fig. S1 for locations of teleseismic events. Here the CRF images only show the signals with a 68% confidence level. From top to bottom, panels show results obtained for different frequency bands. **b** Similar to (a), but for CRF images obtained using short-period data. **c** Similar to (a), but for CRF images obtained using both broadband and short-period data.

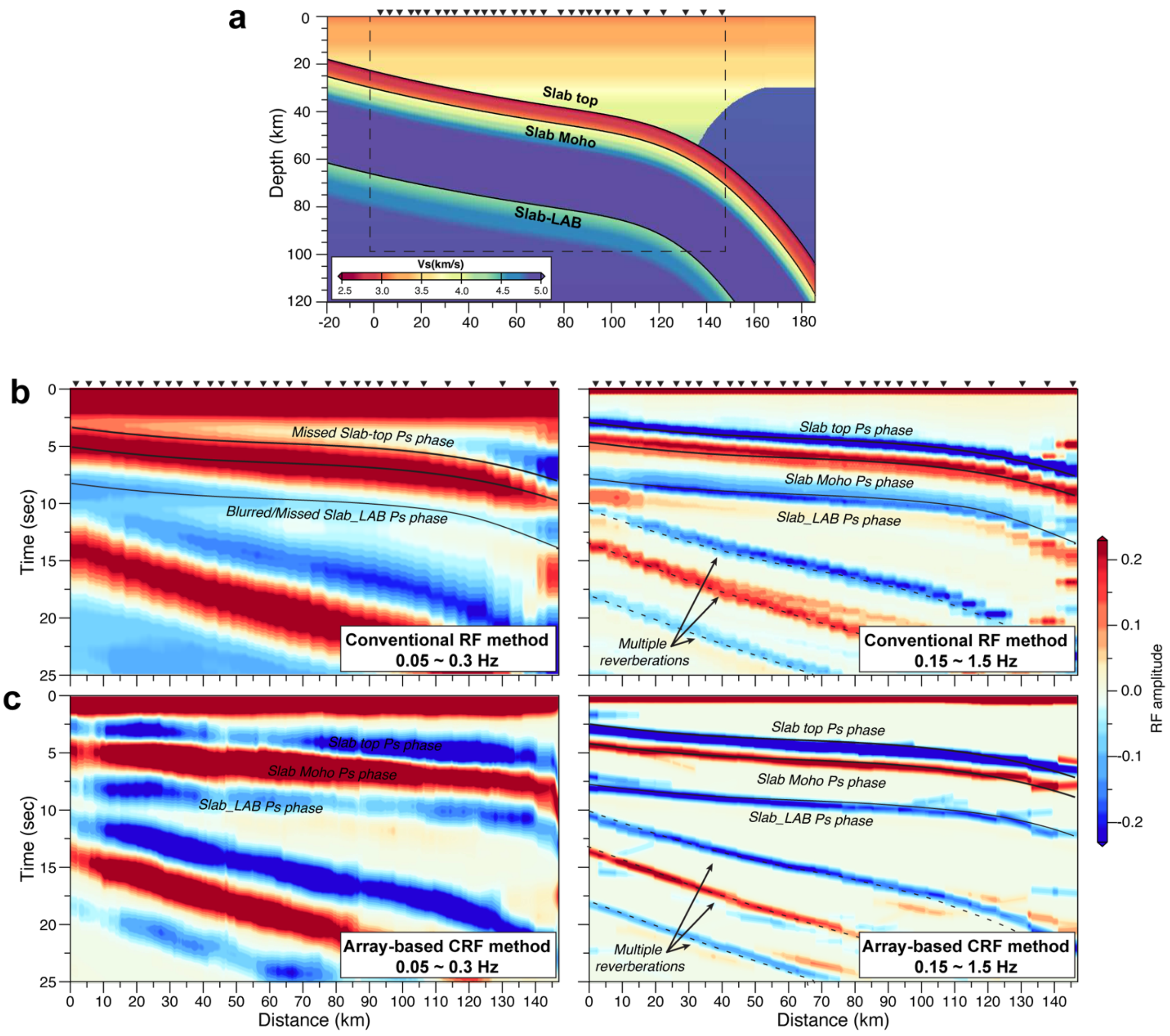

Fig. S7 | Synthetic tests (noise-free) for evaluating the performance of conventional Receiver Function (RF) method and array-based Coherent Receiver Function (CRF) method. a The synthetic subduction zone model consists of the slab top, slab Moho, and slab-LAB discontinuities, as detailed in Fig. 2. Here, our synthetic tests only use the broadband seismic stations, as most previous studies only focused the broadband stations. Synthetic seismograms are generated for the specific source-receiver pairs (focused on the earthquakes located in the southeast direction in Fig. S1) in central Cascadia to simulate real situations. Then, the synthetic data is processed in a similar manner as the real observations, as detailed in Materials and Methods. No noise is added to the synthetic data to isolate the effect of noise on subsurface structure imaging. **b** Conventional RF stacking images. In the low-frequency (0.05–0.3 Hz) of RF image, the signal related to slab-top direct Ps conversion is almost disappeared, and there are only sporadic negative signals related to slab-LAB. **c** Array-based CRF stacking images. The Slab-top and Slab-LAB signals can be well observed in the array-based CRF stacking images, both in low-frequency and high-frequency analyses.

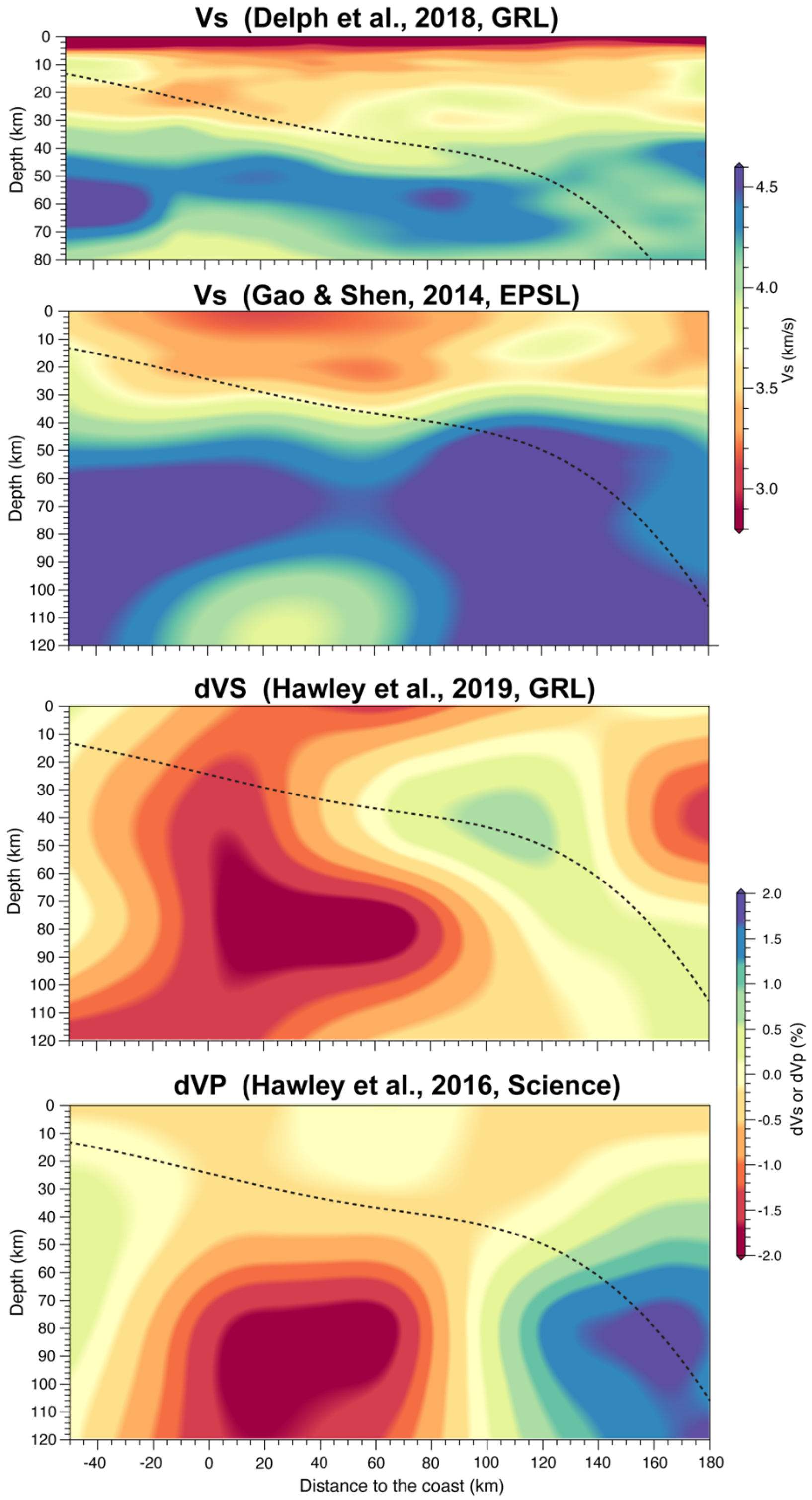

Fig. S8 | Comparison of tomographic models along the seismic array. From the top to bottom, panels show the velocity models from Delph et al.[5], Gao and Shen[6], Hawley and Allen[7], and Hawley et al.[8]. The dashed lines represent the slab interface model of Slab2.0[3].

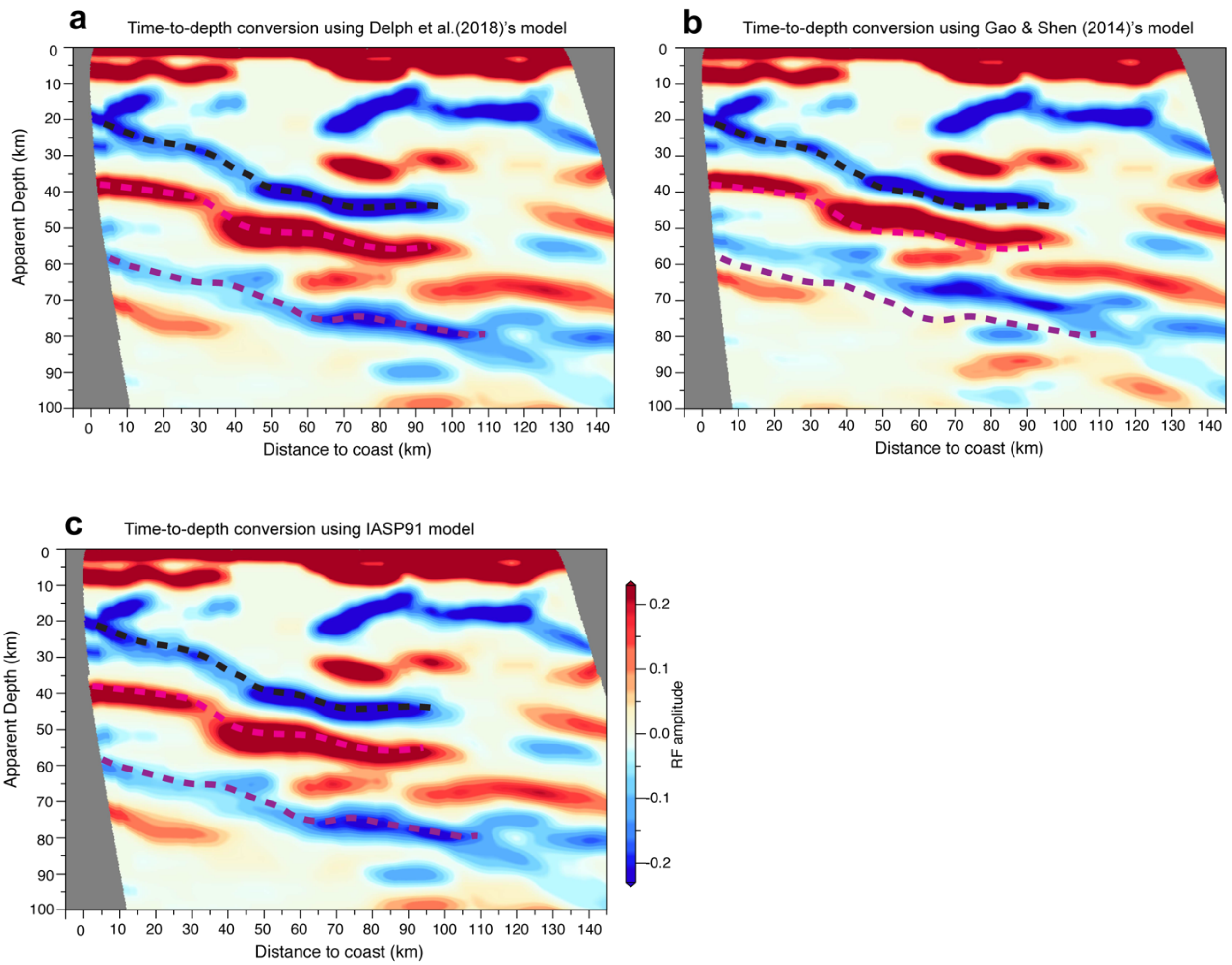

Fig. S9 | Comparison of time-to-depth converted Coherent Receiver Function (CRF) images using different velocity models. a The CRF images obtained using the tomography model of Delph et al.[5] for time-to-depth conversion. **b** The CRF images obtained using the tomography model of Gao and Shen[6] for time-to-depth conversion. **c** The CRF images obtained using 1D IASP91 model[9]. The dashed lines in panel (a) represent three interpreted discontinuities in the main text, and the dashed lines in panels (b) and (c) are the same as those in panel (a) for comparison. The other two tomography models shown in Fig. S8 were not used in time-to-depth conversion, because the original papers only provide velocity anomalies, rather than absolute velocity values. We also examined the trade-off between velocity models and estimated depth in Fig. S10.

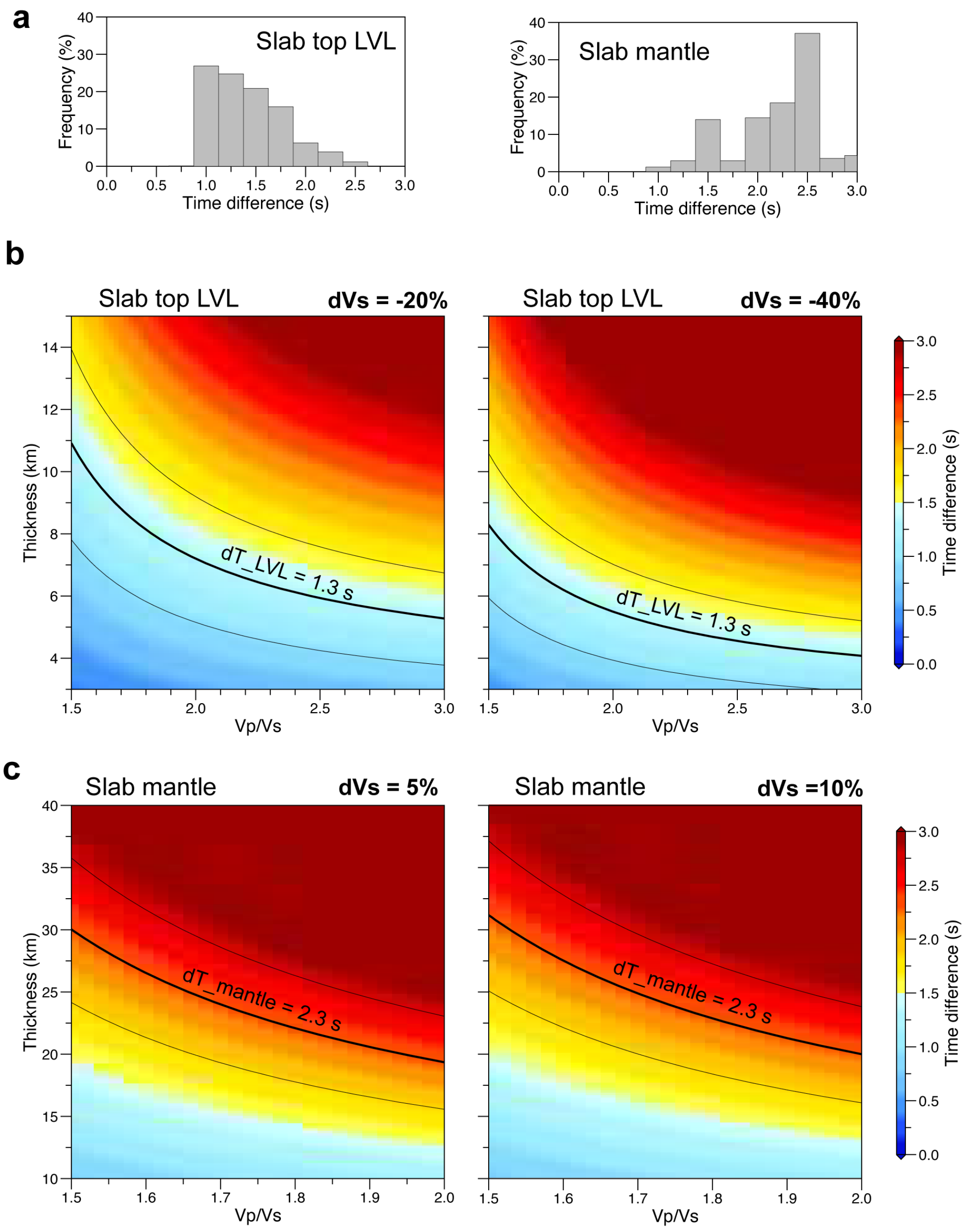

Fig. S10 | Effects of velocity models in estimating the thickness of slab-top low-velocity layer and slab mantle. a The observed arrival time differences between the slab-top interface and slab Moho (left) and the time differences between the slab Moho and slab-LAB (right). **b** The trade-off among the thickness, Vs, and Vp/Vs ratio for the slab-top low-velocity layer (LVL), which is the layer between the slab-top interface and slab Moho. The background figure shows that a strong trade-off exists between thickness estimation and the velocity models used in the time-to-depth conversion. The black thick and dashed lines represent the observed arrival time of slab top LVL and its uncertainties, respectively. **c** Similar to (b), but for the slab mantle, which is the layer between the slab Moho and slab-LAB.

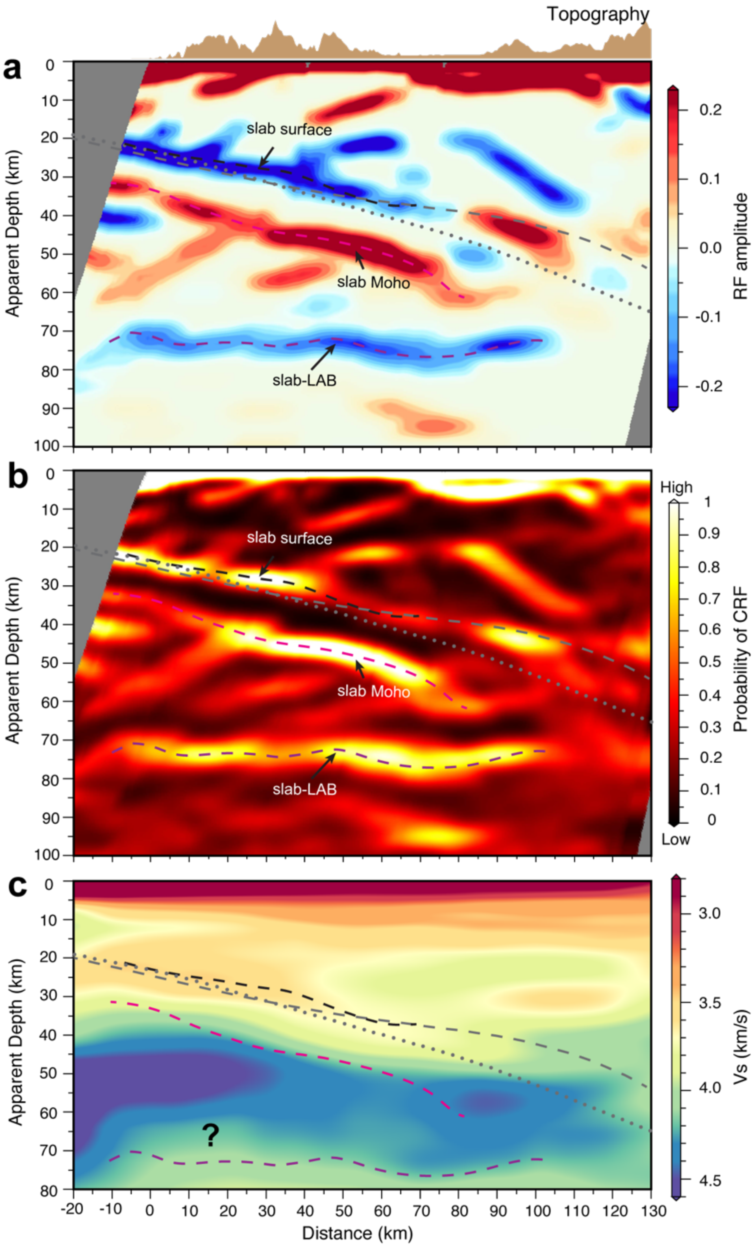

Fig. S11 | Coherent Receiver Function (CRF) images constructed using seismic waves from the northwest direction. a The CRF stacking image. See Fig. S1 for the locations of teleseismic events and Ps piercing points. This image shows the subsurface structure for an area immediately to the north of the linear seismic array, compared to the CRF image shown in Fig. 3 which shows the structures to the south of the linear array (Fig. S1). **b** Density plot of the ensemble solutions that represents the posterior probability distribution of the timing of the CRF phases, which serves as a measure of the reliability of the imaged structures. **c** Tomographic image from Delph et al.[5]. The inferred slab upper interface, slab Moho, and slab-LAB are highlighted by black, red, and purple dashed lines, respectively. The thin dashed and dotted gray lines represent the subduction interface model from the Slab2.0[3] and McCrory et al.[4], respectively. The observed slab-LAB roughly resembles the shape of the high-velocity slab shown in the tomographic model, although it manifests at a depth ~10 km deeper at distances of ~0-40 km along the profile, a result unlikely

due solely to the uncertainties of the velocity model used in the time-to-depth conversion. Further studies are required to understand the 3D slab effects in RF imaging and to incorporate more accurate tomographic models in the time-to-depth migration.

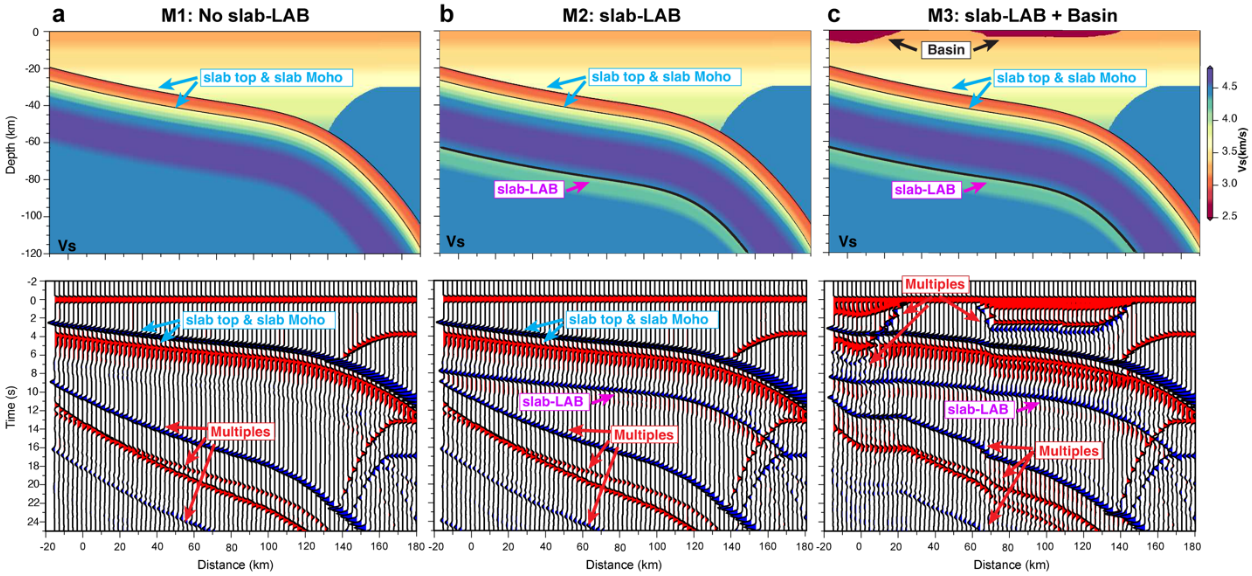

Fig. S12 | Effect of multiple reverberations of shallow structures on slab-LAB imaging. a Model-1 consists of a 7 km thick low-velocity subducting oceanic crust and a 33 km thick high-velocity subducting oceanic mantle, where the transition from the subducting lithosphere to the underlying asthenosphere is smooth. The slab geometry is based on the Slab2.0 model[3] in central Cascadia to mimic real situations. From the top to bottom, panels show the 2D synthetic model and the corresponding synthetic receiver function waveforms. While our CRF images show a >10 km thick subducting oceanic crust in Fig. 3, it is important to note that the thickness estimate may be biased due to the velocity models used in time-to-depth conversion, as mentioned in the main manuscript. Therefore, in our synthetic tests, we used a normal oceanic crust with a thickness of 7 km. If a thicker oceanic crust is employed, it would significantly delay the Moho reverberations and the slab-LAB is still free from these reverberations. **b** Similar to (a), but for the Model 2 that consists of a sharp velocity reduction at the base of the subducting slab. **c** Similar to (a), but for the Model 3 that includes shallow sedimentary basins to test the effect of the basin reverberations on slab-LAB imaging.

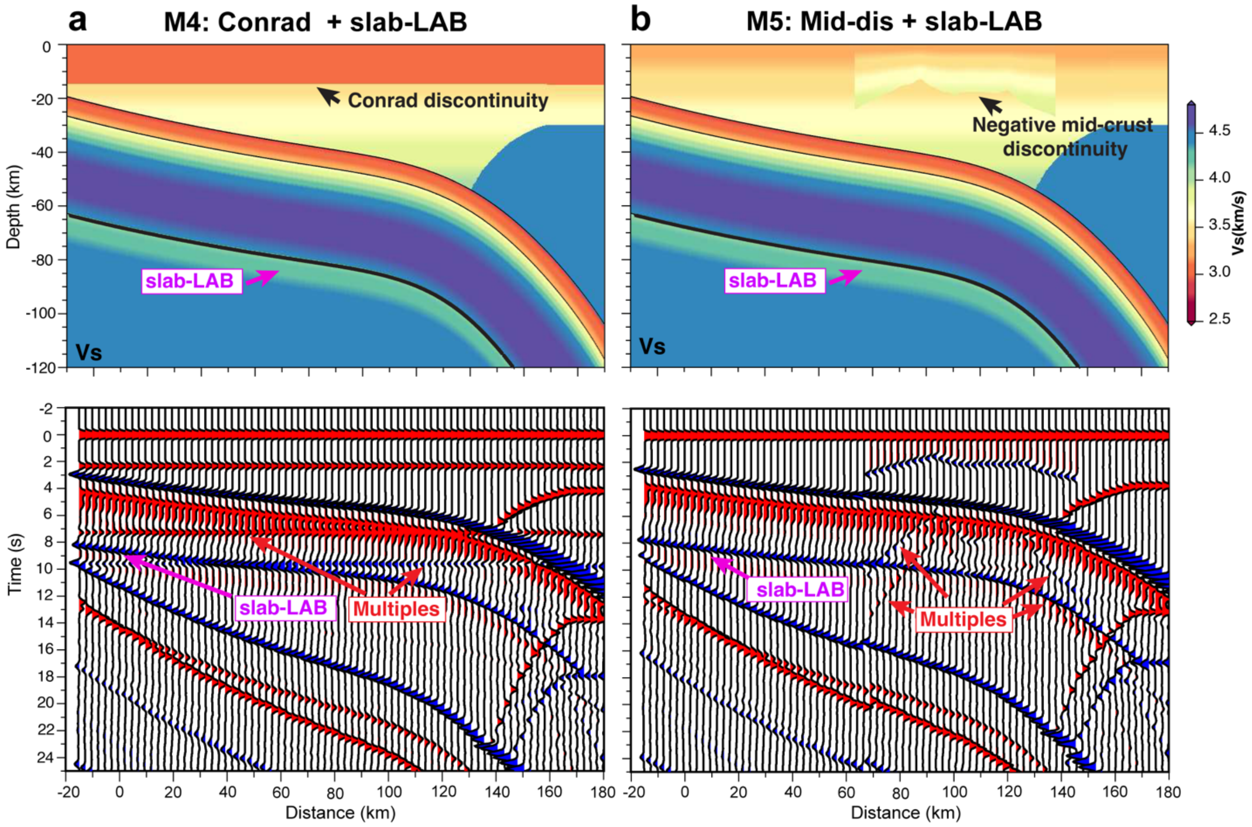

Fig. S13 | Effect of multiple reverberations of an intra-crustal discontinuity on slab-LAB imaging. a Similar to Model 2 shown in Fig. S12, but for the Model 4 that includes the positive Conrad discontinuity. Multiples from an intra-crustal discontinuity would exhibit a pair of positive and negative signals. In addition, the multiples from Conrad discontinuity show different amplitude variations with respect to the incident directions (Fig. S15), and the arrival time of the negative phase decreases with the increasing of epicentral distance (Fig. S16). **b** Similar to Model 2 shown in Fig. S12, but for the Model 5 that includes a negative intra-crustal discontinuity (Fig. 3). The geometry of the multiples is inconsistent with the slab-LAB discontinuity observed in this study.

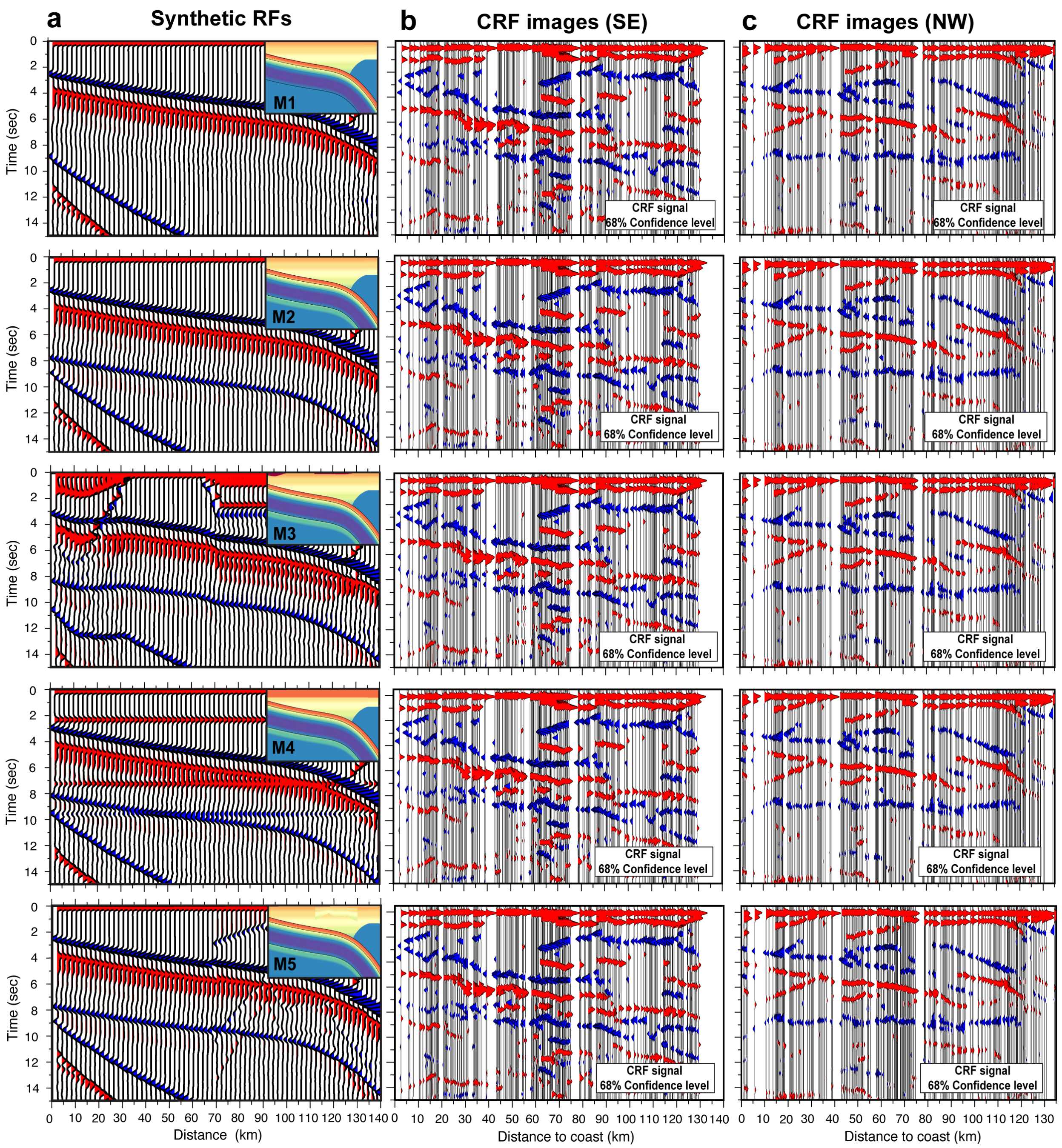

Fig. S14 | Comparison of the synthetic Receiver Function (RF) and Coherent Receiver Function (CRF) images. a The synthetic RFs, similar to that shown in Fig. S12 but with the same time (depth) and distance scale with the real observations. **b** The CRF images with seismic waves from the southeast direction. **c** The CRF images with seismic waves from the northwest direction.

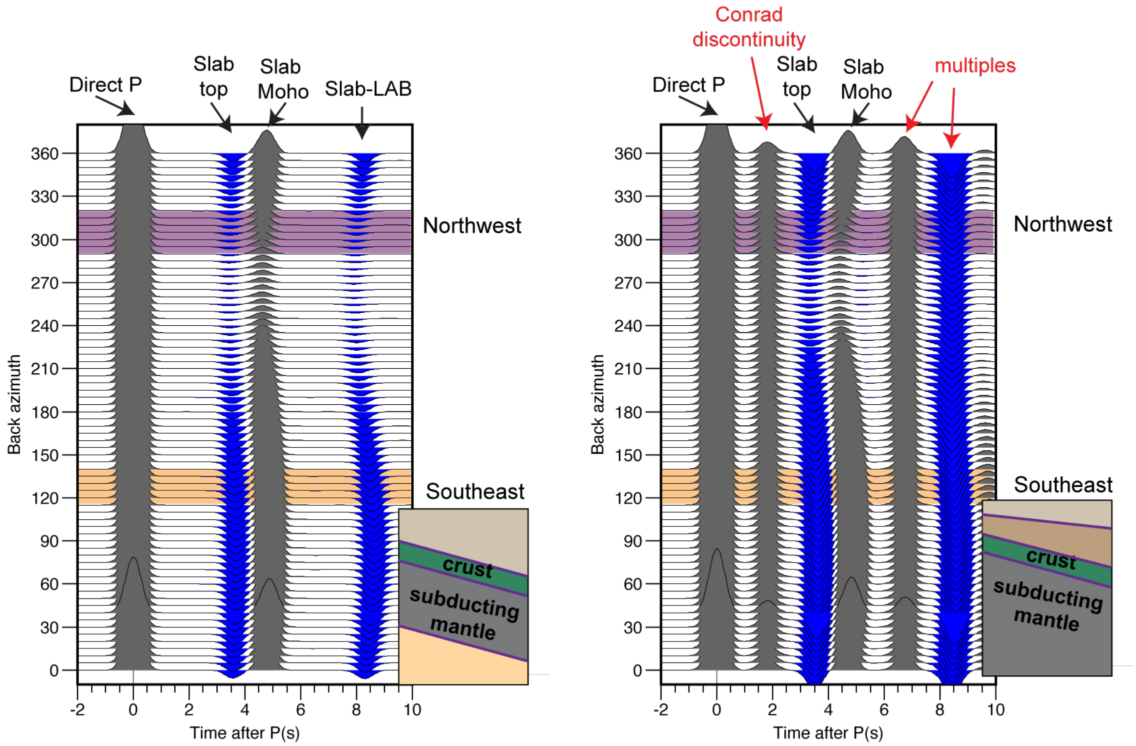

Fig. S15 | Comparison of synthetic Receiver Function (RF) waveforms as a function of back-azimuths generated by two different models: the slab-LAB model and the Conrad discontinuity model. A key difference between the RFs generated by the two models is whether there is a positive phase just before the negative phase. In addition, the multiples of the Conrad discontinuity and the direct Ps conversions of the slab-LAB exhibit distinct variations in terms of amplitude in back-azimuth. The three highlighted strips represent the three earthquake groups shown in Fig. S1. As shown in Fig. S4, the RF images constructed with seismic waves from the northwest shows a much weaker negative discontinuity compared to those obtained using the waveforms from the southeast direction, suggest that the negative phase is related to the direct Ps phase rather than the multiples.

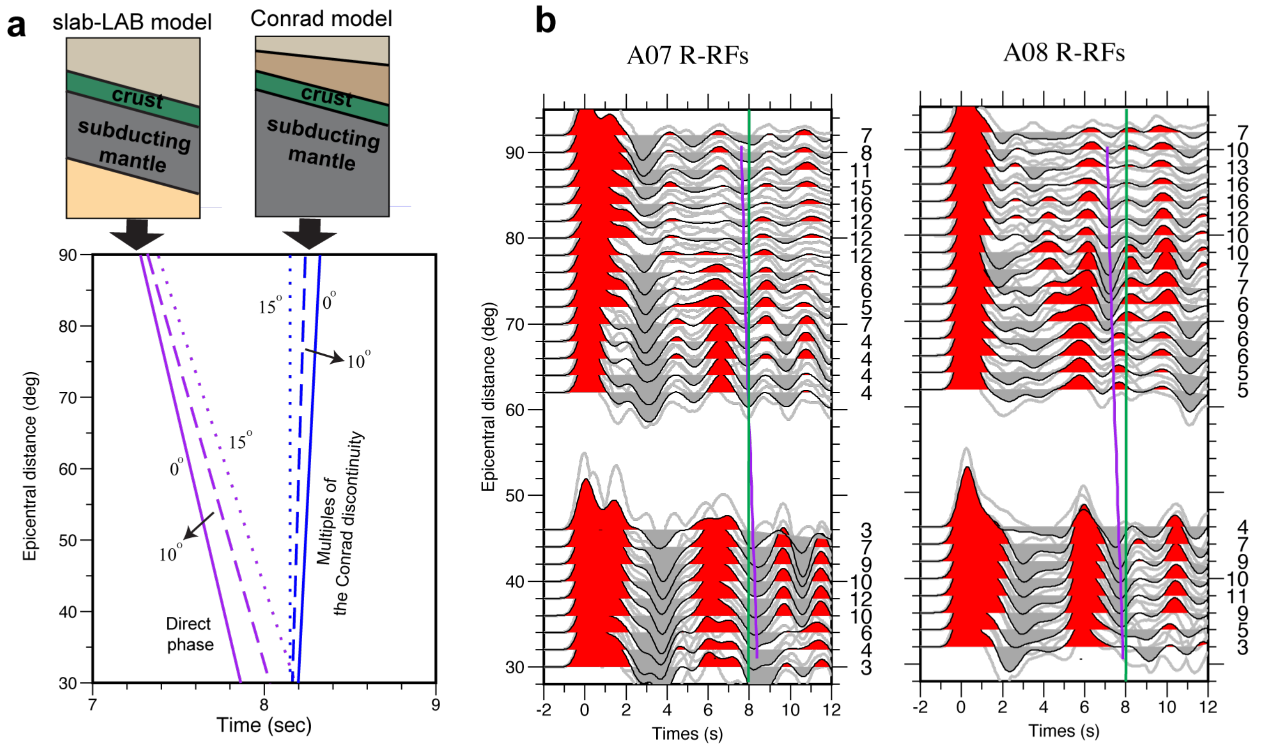

Fig. S16 | Comparison of Receiver Function (RF) arrivals as a function of earthquake epicentral distance generated by two different models: the slab-LAB model and the Conrad discontinuity model. a Synthetic RF arrival times as a function of earthquake epicentral distance. The purple lines show the arrival times of the direct Ps conversion of the slab-LAB discontinuity, while the blue lines indicate the arrival times of the multiples of the Conrad discontinuity. We also tested the dipping Conrad discontinuity. The numbers displayed around the lines correspond to the dip angle of the discontinuity in the synthetic modeling. The direct Ps phases exhibit negative slowness (arrival time decrease with increasing epicentral distance) due to their steeper incidence angle. In contrast, the multiples follow a positive slowness moveout due to a shallower incidence angle. **b** Real RF observations at two representative stations. The RF waveforms have been stacked by epicenter distance using 5° bins with an increment of 2°. The number of stacked traces in each bin is indicated on the right side. The negative phases around 8 seconds show a negative slowness (highlighted in purple lines), suggesting that the negative phase is related to the direct Ps phase rather than the multiples.

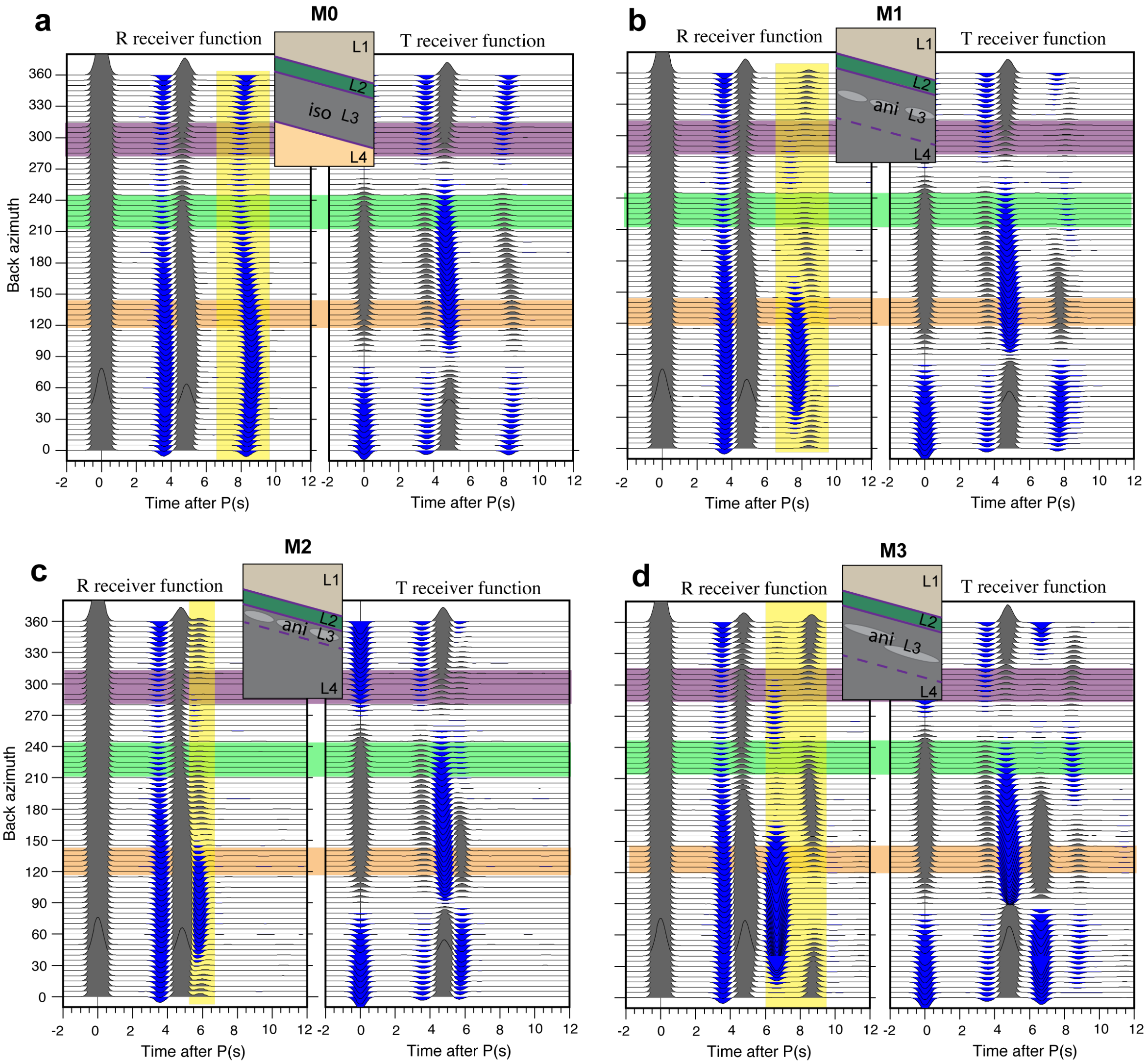

Fig. S17 | Effect of a dipping interface and anisotropy layer on Receiver Function (RF). a Slab-LAB model. The input model includes a 7 km oceanic crust and a 30 km thick high-velocity subducting oceanic mantle. The slab geometry is based on the Slab2.0 model[3] in central Cascadia to mimic real situations. The seismic discontinuities are highlighted with purple lines. The slab-LAB Ps conversion appears as a negative phase with arrivals that vary in a period of 2π with the back-azimuths. **b** Anisotropy model. The model consists of a 30 km thick anisotropy (10% strength) layer within the subducted oceanic mantle. The trend and plunge of the fast axis are set based on previous studies and local geological settings to represent the fossil fabric generated at spreading ridges. Depending on the back-azimuth of arriving waves, the anisotropy model would result in either a positive or negative Ps phase, with the Ps arrivals varying in a period of π with the back-azimuth. **c** Anisotropy model (M2) with a 10 km thick anisotropy (10% strength) layer within the subducted oceanic mantle. **d** Anisotropy model (M3) with a 30 km thick anisotropy (25% strength) layer within the subducted oceanic mantle. Details of the model setup in synthetic tests are provided in Table S1. The three horizontal strips highlight the three groups of earthquakes in the northwest, southwest, and southeast directions shown in Fig. S1. The vertical yellow strips highlight the Ps conversions related to the dipping slab-LAB or anisotropy layer.

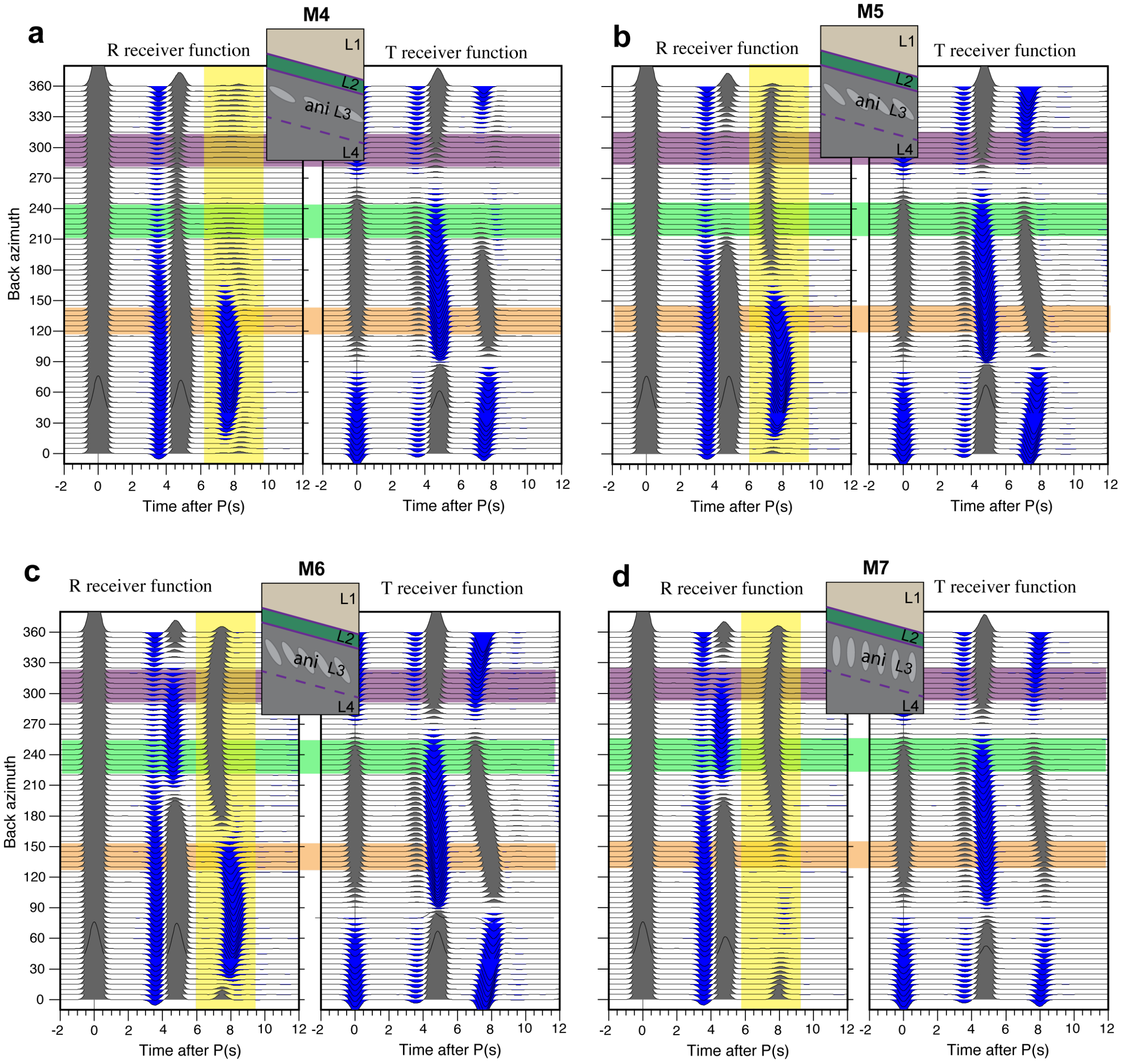

Fig. S18 | Effect of the plunge of the symmetry axis of the anisotropy layer on Receiver Function (RF). a Anisotropy model (M4) with a 30 km thick anisotropy layer (10% strength with a plunge angle of 30°) within the subducted oceanic mantle. **b** Anisotropy model (M5) similar to a, but with a plunge angle of 45°. **c** Anisotropy model (M6) similar to a, but with a plunge angle of 60°. **d** Anisotropy model (M7) similar to a, but with a plunge angle of 90°. Details of the model setup in synthetic tests are provided in Table S1. The three horizontal strips highlight the three groups of earthquakes in the northwest, southwest, and southeast directions shown in Fig. S1. The vertical yellow strips highlight the Ps conversions related to the dipping slab-LAB or anisotropy layer.

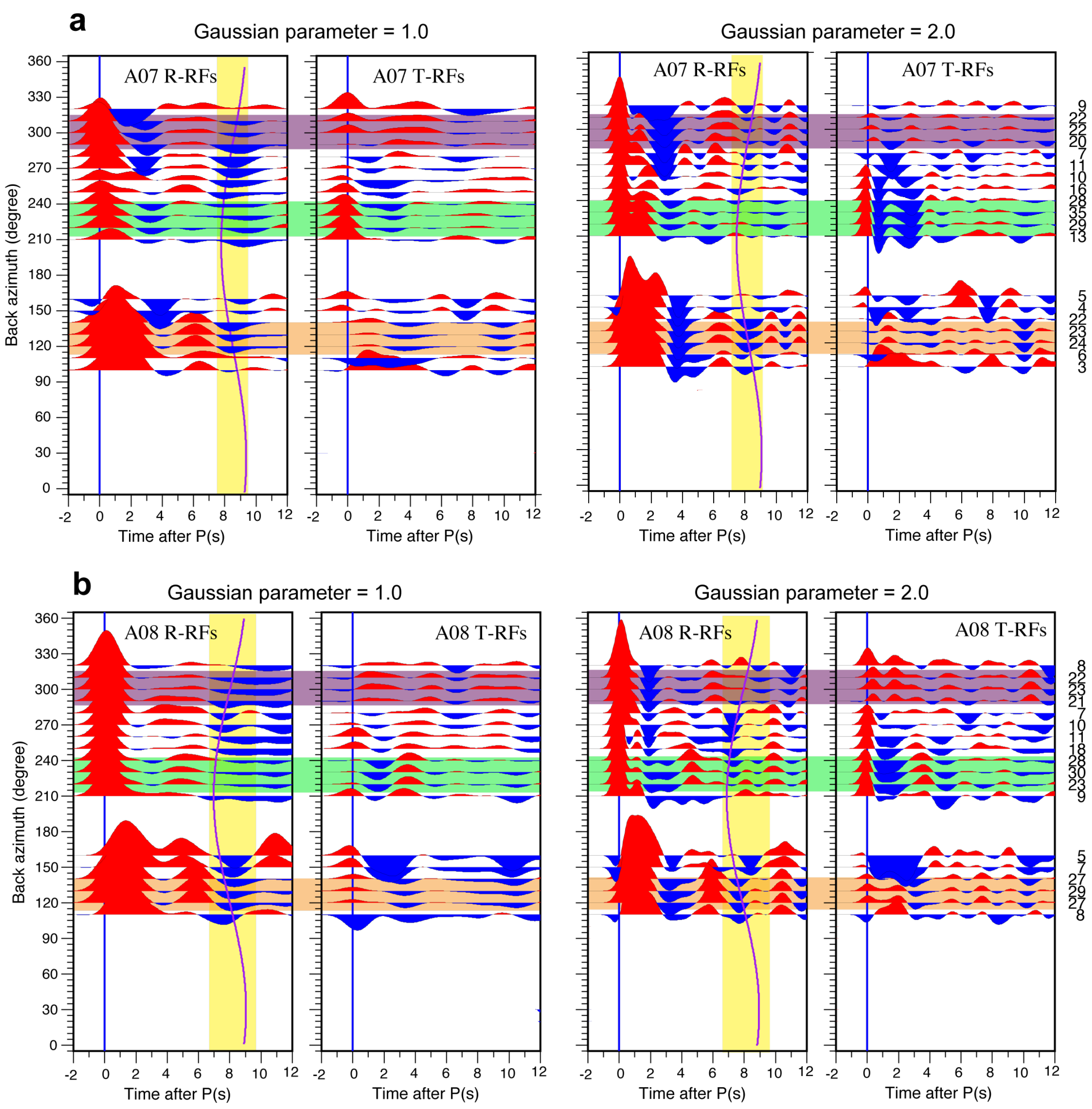

Fig. S19 | Observed radial and tangential Receiver Functions (RFs) for broadband stations plotted as a function of back-azimuths. a The A07 station. **b** The A08 station. The RF waveforms have been stacked by back-azimuth using 15° bins with an increment of 10°. The number of stacked traces in each bin is indicated on the right side. The negative phase following the slab-Moho, highlighted in yellow, is better explained by a period of 2π pattern, with nearly all negative phases across all back-azimuths. The real observations are better explained by a dipping interface model, as compared to the synthetics shown in Figs. S17 and S18.

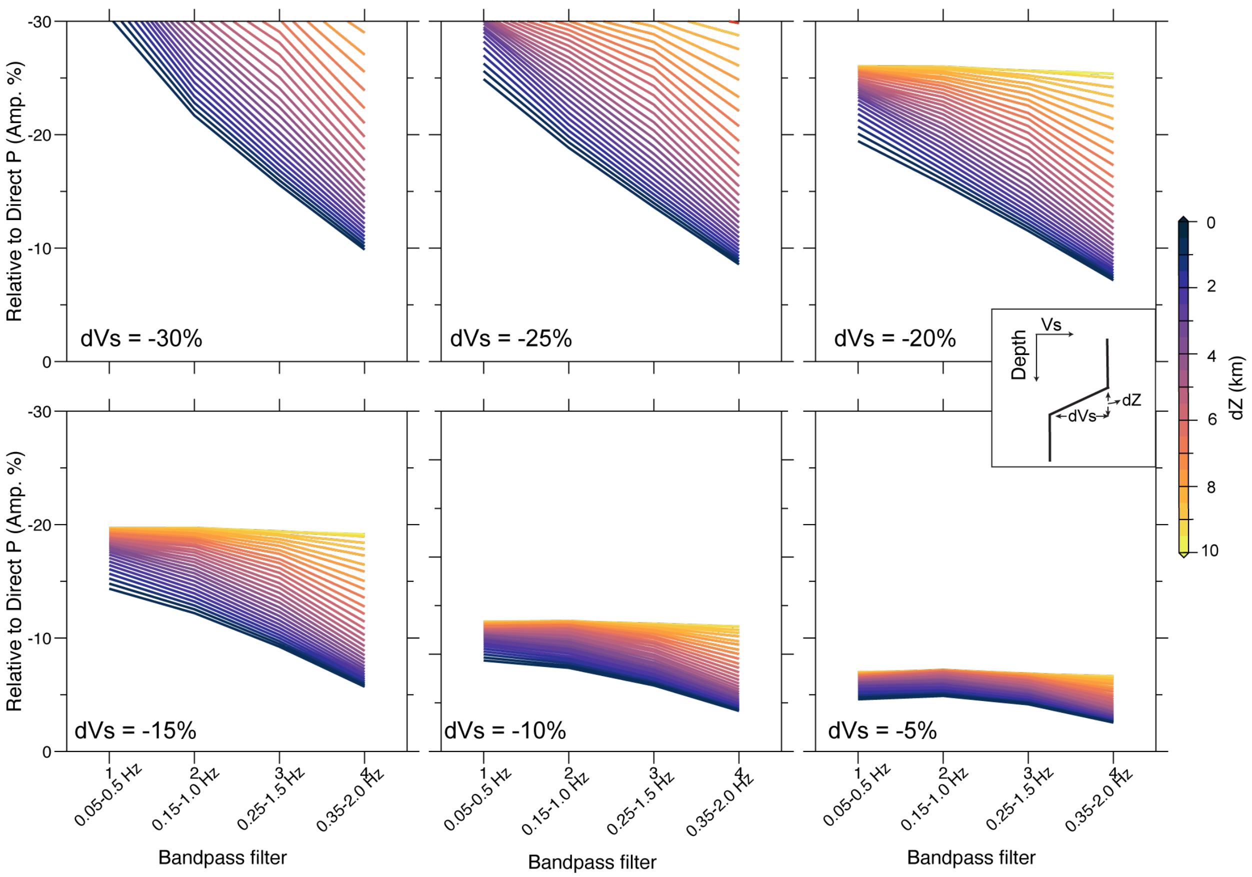

Fig. S20 | Diagrams showing the variation of the Ps amplitude as a function of the velocity decrease (dVs), the depth range (dZ) over which the decrease occurs, and the frequency band used in analyses.

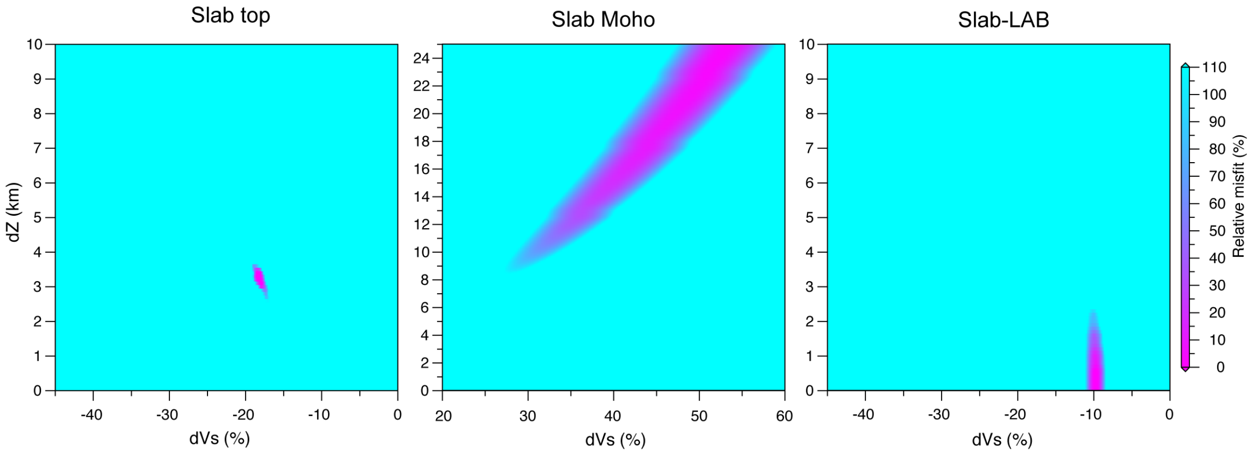

Fig. S21 | Misfits as a function of the velocity decrease (dVs), the depth range (dZ) over which the decrease occurs. Both the dVs and dZ are well constrained for the slab top and slab-LAB, but there is a large tradeoff between the two for the slab Moho. The slab Moho is not well constrained partly because the analysis of the Ps phase at low frequencies cannot distinguish the slab Moho from the broader gradient of high-velocity subducting slab mantle.

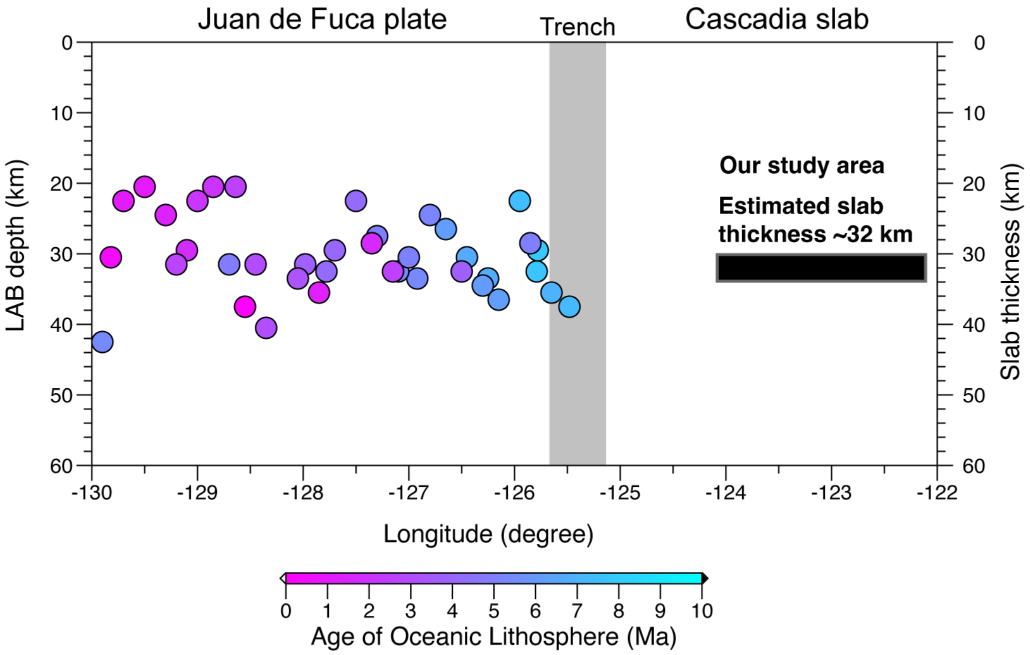

Fig. S22 | Comparison of the Juan de Fuca plate-LAB before subduction and slab-LAB after subduction. The depths of the Juan de Fuca plate-LAB (data from Rychert et al.[10]), color-coded by the age of oceanic lithosphere[11], are for the oceanic area within the latitude range of 43°N-46°N to the west of our study region (~44.4°N). The thickness of the Cascadia slab is estimated from this study based on the depths of the slab surface and slab-LAB. The estimated slab thickness from our study is consistent with the depth of the Juan de Fuca Plate-LAB before subduction.

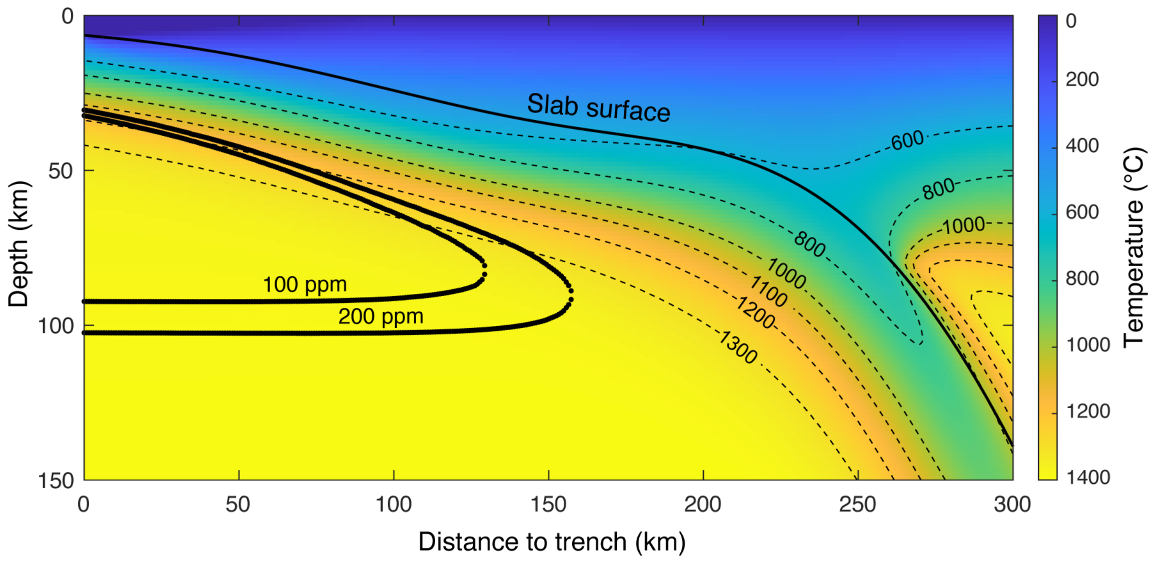

Fig. S23 | Geothermal model[12] in Cascadia and the corresponding mantle solidus calculated using different amounts of water[13].

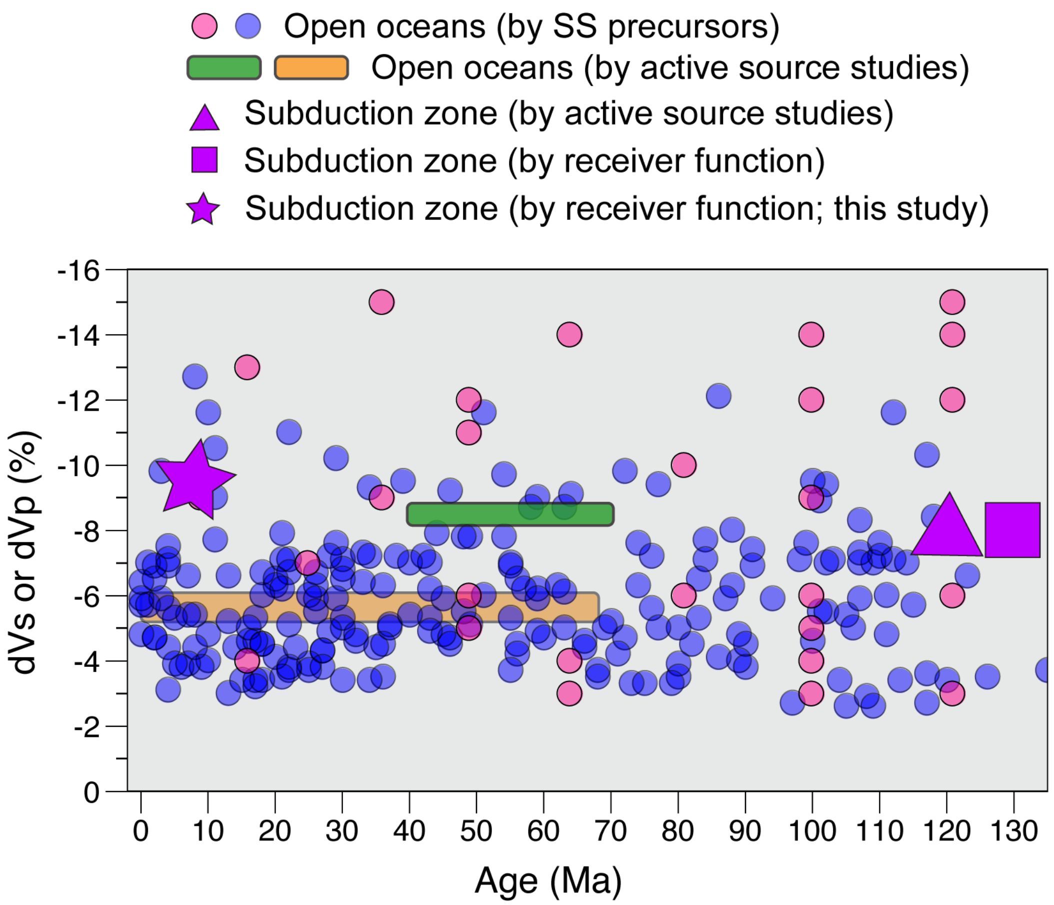

Fig. S24 | Global observations of sharp seismic velocity reduction at the base of oceanic lithosphere beneath oceanic plates in open ocean and subducting slabs in subduction zones. The blue and red circles indicate results beneath the Pacific ocean from SS precursor studies by Schmerr[14] and Tharimena et al.[15], respectively. The green and orange bars indicate a relatively constant Vp reduction from active-source seismic studies in the Atlantic Ocean[16,17]. The purple star, triangle, and square show the results in subduction zones from our study, Stern et al.[18], and Kawakastu et al.[19], respectively.

Table S1.
Model parameters in modeling dipping interface and anisotropy layer.

		Thick [km]	Vp [km/s]	Vs [km/s]	Vp_ani [%]	Vs_ani [%]	Trend [deg]	Plunge [deg]	Strike [deg]	Dip [deg]
**Dipping model (M0)**	Layer-1	30	6.5	3.5	-	-	-	-	-	-
	Layer-2	7	6.0	3.1	-	-	-	-	356	15
	Layer-3	30	7.9	4.3	-	-	-	-	356	15
	Layer-4	-	7.2	3.8	-	-	-	-	-	-
**Anisotropy Model** (M1)	Layer-1	30	6.5	3.5	-	-	-	-	-	-
	Layer-2	7	6.0	3.1	-	-	-	-	356	15
	Layer-3	30	7.9	4.3	10	10	90	15	356	15
	Layer-4	-	7.9	4.3	-	-	-	-	-	-
**Anisotropy Model** (M2)	Layer-1	30	6.5	3.5	-	-	-	-	-	-
	Layer-2	7	6.0	3.1	-	-	-	-	356	15
	Layer-3	10	7.9	4.3	10	10	90	15	356	15
	Layer-4	-	7.9	4.3	-	-	-	-	-	-
**Anisotropy Model** (M3)	Layer-1	30	6.5	3.5	-	-	-	-	-	-
	Layer-2	7	6.0	3.1	-	-	-	-	356	15
	Layer-3	30	7.9	4.3	25	25	90	15	356	15
	Layer-4	-	7.9	4.3	-	-	-	-	-	-
**Anisotropy Model** (M4)	Layer-1	30	6.5	3.5	-	-	-	-	-	-
	Layer-2	7	6.0	3.1	-	-	-	-	356	15
	Layer-3	30	7.9	4.3	10	10	90	30	356	15
	Layer-4	-	7.9	4.3	-	-	-	-	-	-
**Anisotropy Model** (M5)	Layer-1	30	6.5	3.5	-	-	-	-	-	-
	Layer-2	7	6.0	3.1	-	-	-	-	356	15
	Layer-3	30	7.9	4.3	10	10	90	45	356	15
	Layer-4	-	7.9	4.3	-	-	-	-	-	-
**Anisotropy Model** (M6)	Layer-1	30	6.5	3.5	-	-	-	-	-	-
	Layer-2	7	6.0	3.1	-	-	-	-	356	15
	Layer-3	30	7.9	4.3	10	10	90	60	356	15
	Layer-4	-	7.9	4.3	-	-	-	-	-	-
**Anisotropy Model** (M7)	Layer-1	30	6.5	3.5	-	-	-	-	-	-
	Layer-2	7	6.0	3.1	-	-	-	-	356	15
	Layer-3	30	7.9	4.3	10	10	90	90	356	15
	Layer-4	-	7.9	4.3	-	-	-	-	-	-

References:

1. Wessel, P. *et al.* The Generic Mapping Tools Version 6. *Geochem. Geophys. Geosyst.* **20**, 5556–5564 (2019).

2. Ryan, W. B. F. *et al.* Global Multi-Resolution Topography synthesis. *Geochem. Geophys. Geosyst.* **10**, (2009).

3. Hayes, G. P. *et al.* Slab2, a comprehensive subduction zone geometry model. *Science* **362**, 58–61 (2018).

4. McCrory, P. A., Blair, J. L., Oppenheimer, D. H. & Walter, S. R. Depth to the Juan de Fuca slab beneath the Cascadia subduction margin: A 3-D model for sorting earthquakes. *US Geol. Surv. Dig. Data Ser*. 91 (2006).

5. Delph, J. R., Levander, A. & Niu, F. Fluid controls on the heterogeneous seismic characteristics of the Cascadia margin. *Geophys. Res. Lett.* **45**, 11–021 (2018).

6. Gao, H. & Shen, Y. Upper mantle structure of the Cascades from full-wave ambient noise tomography: Evidence for 3D mantle upwelling in the back-arc. *Earth Planet. Sci. Lett.* **390**, 222–233 (2014).

7. Hawley, W. B. & Allen, R. M. The Fragmented Death of the Farallon Plate. *Geophys. Res. Lett.* **46**, 7386–7394 (2019).

8. Hawley, W. B., Allen, R. M. & Richards, M. A. Tomography reveals buoyant asthenosphere accumulating beneath the Juan de Fuca plate. *Science* **353**, 1406 (2016).

9. Kennett, B. L. N. & Engdahl, E. R. Traveltimes for global earthquake location and phase identification. *Geophys. J. Int.* **105**, 429–465 (1991).

10. Rychert, C. A., Harmon, N. & Tharimena, S. Scattered wave imaging of the oceanic plate in Cascadia. *Sci. Adv.* **4**, eaao1908 (2018).

11. Seton, M. *et al.* A Global Data Set of Present-Day Oceanic Crustal Age and Seafloor Spreading Parameters. *Geochem. Geophys. Geosyst.* **21**, e2020GC009214 (2020).

12. Abers, G. A., van Keken, P. E. & Wilson, C. R. Deep decoupling in subduction zones: Observations and temperature limits. *Geosphere* **16**, 1408–1424 (2020).

13. Katz, R. F., Spiegelman, M. & Langmuir, C. H. A new parameterization of hydrous mantle melting. *Geochem. Geophys. Geosyst.* **4**, (2003).

14. Schmerr, N. The Gutenberg Discontinuity: Melt at the Lithosphere-Asthenosphere Boundary. *Science* **335**, 1480–1483 (2012).

15. Tharimena, S., Rychert, C., Harmon, N. & White, P. Imaging Pacific lithosphere seismic discontinuities—Insights from SS precursor modeling. *J. Geophys. Res. Solid Earth* **122**, 2131–2152 (2017).

16. Mehouachi, F. & Singh, S. C. Water-rich sublithospheric melt channel in the equatorial Atlantic Ocean. *Nat. Geosci.* **11**, 65–69 (2018).

17. Audhkhasi, P. & Singh, S. C. Discovery of distinct lithosphere-asthenosphere boundary and the Gutenberg discontinuity in the Atlantic Ocean. *Sci. Adv.* **8**, eabn5404 (2022).

18. Stern, T. A. *et al.* A seismic reflection image for the base of a tectonic plate. *Nature* **518**, 85 (2015).

19. Kawakatsu, H. *et al.* Seismic Evidence for Sharp Lithosphere-Asthenosphere Boundaries of Oceanic Plates. *Science* **324**, 499–502 (2009).
